# Supplementary material for: PrEP Scale-Up and PEP in Central and Eastern Europe: Changes in Time and the Challenges We Face with No Expected HIV Vaccine in the near Future
Source: Vaccines (Basel). 2023 Jan 4;11(1):122. doi: 10.3390/vaccines11010122 (PMC9867039; doi:10.3390/vaccines11010122)
Supplement: Supplementary file 1 [file vaccines-11-00122-s001.zip › vaccines-2101285-supplementary.pdf]

Dear Colleagues, the aim of this questionnaire is to present a snapshot of pre- and post-exposure prophylaxis (PrEP and PEP) in Central and Eastern Europe. Please respond to the questions considering the general overview of your country in terms of PrEP and PEP. Please mark only one item if not stated otherwise.

Thank you for your cooperation.

Is TDF/FTC currently licensed by drug registration authority for the use in HIV negative persons in your country?

1. Yes
2. No
3. Don't know

If yes, please choose the PrEP type licensed.

1. On demand
2. Daily
3. Both
4. Other

Do you have PrEP recommended in national guidelines?

1. Yes-in HIV guidelines
2. No - we have national guidelines, but they don't mention PrEP
3. No - we don't have national guidelines
4. Other

Do you use any other PrEP guidelines?

1. No
2. Yes

Is PrEP prescribed in your country?

1. Yes - free of charge within public health
2. Yes - within public health, but patient needs to pay for TDF/FTC
3. Yes - within public health, but patient needs to pay for some tests and TDF/FTC
4. Yes - but only in private practices (all costs fully paid by the patient)
5. No
6. Other

Are you prescribing PrEP?

1. Yes in public practice
2. Yes in private practice
3. No
4. Other

Is PrEP available to buy at the pharmacies in your country?

1. Yes, original Truvada
2. Yes, generic TDF/FTC

3. Yes, both generic and original
4. No
5. Other availability

If yes for generic TDF/FTC, what is the cost of one package in Euros?

If yes, for original Truvada, what is the cost of one package in Euros?

Are you aware of ‘informal’ PrEP use in your country/centre (eg. through friends or bought online)?

1. Yes
2. No

How many PrEP offering centres do you have in your country?

How many people on PrEP do you estimate to have in your country?

How many people on PrEP do you estimate to have in your center?

How many new HIV infections did you have in your country in 2020?

How many newly diagnosed HIV patients did you have in your center in 2020?

Do you ask newly diagnosed HIV patients, if they have used PrEP?

1. Yes
2. No

How many newly diagnosed patients in your centre had used PrEP prior to their HIV diagnosis?

What is your estimation for the access rate to PrEP in your country (average %)?

What do you think the obstacles to access/wider access to PrEP is? (please mark all relevant items)

1. Not licensed
2. Not reimbursed
3. Lack of knowledge/awareness by people in need
4. Low perception of HIV risk
5. Lack of knowledge/awareness by healthcare providers
6. Fear of stigma and discrimination
7. Fear of side effects
8. Lack of decentralization
9. Other (please specify)

Are there any clinical trials with PrEP currently available in your country (eg. Long-acting injectables or new strategies for oral PrEP)?

1. Yes
2. No

If yes to above, please specify

Is non-occupational post-exposure prophylaxis available in your country?

1. Yes
2. No

If yes do you have a national guideline for non-occupational PEP?

1. Yes
2. No

Is non-occupational PEP fully reimbursed?

1. Yes, without restrictions
2. Yes, but only for accidental exposure (e.g. sex assault, accidental needle stick not related to occupation)
3. Yes, but only for pre-defined groups (eg. sex workers, anal sex); specify which:
4. No

Is occupational PEP available in your country?

1. Yes
2. No

How is occupational PEP financed in your country (the cost of visit, tests and antiretrovirals)?

1. By general insurance
2. By additional insurance
3. By employer
4. It is not covered
